# High Doses of Norfloxacin Nicotinate Induce Apoptosis, Developmental Neurotoxicity, and Aberrant DNA Methylation in Zebrafish (*Danio rerio*) Larvae

**DOI:** 10.3390/ani16010018

**Published:** 2025-12-20

**Authors:** Hansun Fang, Runping Wang, Fang Wang, Kaibin Li, Huili Liang, Tian Su, Lili Wei, Jiming Ruan, Fugui Li, Ximei Liang

**Affiliations:** 1 Key Laboratory of Poyang Lake Basin Agricultural Resource and Ecology of Jiangxi Province, College of Land Resource and Environment, Jiangxi Agricultural University, Nanchang 330045, China; fanghansun@163.com; 2 College of Animal Science and Technology, Jiangxi Agricultural University, Nanchang 330045, China; wrp201314@126.com (R.W.); hbliliwei@163.com (L.W.); rjm903@163.com (J.R.); 3 Pearl River Fisheries Research Institute, Chinese Academy of Fishery Sciences, Guangzhou 510380, China; wangfang3612@163.com (F.W.); likaibins@126.com (K.L.); lianghuili82@163.com (H.L.); 4 Jiangxi Center Station of Irrigation Experiment, Nanchang 330201, China; leishist@163.com

**Keywords:** zebrafish, norfloxacin nicotinate, apoptosis, DNA methylation, developmental neurotoxicity

## Abstract

This study aimed to evaluate the response mechanisms of zebrafish larvae to Norfloxacin nicotinate (NOR-N) exposure. Embryos were exposed to NOR-N from 4 h post-fertilization (hpf) until 96 hpf. The exposure concentrations included 0.002, 0.2, 1, and 5 mg/L (simulating both normal and exceptionally high environmentally relevant levels of NOR), as well as a high dose of 25 mg/L. Subsequent analyses focused on apoptosis, neurodevelopment, and DNA methylation in the resulting zebrafish larvae. The results showed that high-dose NOR-N (≥5 mg/L) induced obvious apoptotic cell death in zebrafish larvae, accompanied by increased activities of Cas3 and Cas9, up-regulated *P53*, *Bax*, *Puma*, *Apaf1*, *Cas3* and *Cas9* genes expression, and reduced *Mdm2* levels and *Bcl2/Bax* ratio. Moreover, exposure to 5 and/or 25 mg/L NOR-N resulted in a significant up-regulation of neurodevelopment-related genes (*Sox2*, *Sox3* and *Sox19a*), concomitantly with a marked decline in the transcription of DNA methylation genes, including *Dnmt1*, *Dnmt3a1*, *Dnmt3b1*, *Dnmt3b2* and *Dnmt3b4*. Overall, our findings demonstrated that NOR-N exposure could induce apoptosis, developmental neurotoxicity and aberrant DNA methylation in zebrafish larvae. These findings provide insights to guide the safe application of NOR-N in aquaculture and support the assessment of its potential ecological risks to aquatic ecosystems.

## Simple Summary

Norfloxacin nicotinate (NOR-N), a derivative and effective substitute of norfloxacin (NOR), has been widely used in livestock production and aquaculture sectors. However, the adverse impacts of NOR-N on non-target animals and their associated biological response mechanisms are still not completely elucidated. This study revealed that exposure to the high doses of NOR-N induced apoptosis, developmental neurotoxicity and aberrant DNA methylation in zebrafish (*Danio rerio*) larvae. These findings contribute to uncovering the response mechanisms of fish to NOR-N exposure and enhancing understanding of the potential hazards that NOR-N may pose to aquaculture ecosystems.

## 1. Introduction

Norfloxacin (NOR), a broad-spectrum antibacterial fluoroquinolone (FQ), is widely used in human and veterinary medical fields, and also serves as a growth promoter in livestock farming and aquaculture practices [1]. However, due to its adverse effects and potential environmental risks to organisms and human health, the use of NOR has been restricted in aquaculture and livestock husbandry in China [2]. Despite these regulations, structurally related alternatives continue to be introduced into the market. One such compound is norfloxacin nicotinate (NOR-N), a chemical adduct formed from nicotinic acid and NOR, which exhibits superior water solubility and bioavailability compared to NOR [3]. These properties have facilitated its widespread use as a replacement for NOR in aquaculture and livestock production. The standard therapeutic regimen for NOR-N in fisheries involves oral administration at 15–30 mg/kg body weight (calculated as NOR) over 3 to 5 days [4]. Nevertheless, upon entering the bloodstream, NOR-N rapidly dissociates into NOR and nicotinic acid, ultimately exerting its biological effects and undergoing metabolism in the form of NOR [5]. Hence, NOR has been used as the target analyte in studies investigating the pharmacokinetics of NOR-N in animal blood and tissues [3,6]. Consequently, the extensive application of NOR-N has raised concerns regarding the accumulation of NOR in aquatic ecosystems, as well as the associated risks of harm to non-target biota such as fish.

Recent studies have detected NOR residues in global surface water bodies, with concentrations typically spanning the ng/L to µg/L range. In China, levels measured in major river basins fall between 0.03 and 1.38 µg/L, while those in shallow groundwater range from 0.44 to 35.3 μg/L [7]. Even higher concentrations have been reported in other regions, including 52.60 μg/L in Kenya [8] and 470 μg/L in India [9]. Notably, an exceptionally high concentration of 6.06 mg/L was documented in mangrove-associated shrimp aquaculture ponds in Vietnam [10]. While such environmental levels may fall below thresholds of immediate concern for human health, they can nonetheless induce adverse effects in non-target aquatic organisms. Accumulating evidence further indicates that NOR exposure induces multifaceted toxic effects on fish species [11,12,13,14]. For instance, NOR has been shown to induce oxidative stress by altering antioxidative enzyme activities including glutathione peroxidase (Gpx), catalase (CAT) and superoxide dismutase (SOD) in species including male goldfish (0.4–10 mg/L) [15], juvenile zebrafish (*Danio rerio*) (0.0001–30 mg/L) [16] and catfish (0.0025–160 mg/L) [13]. Moreover, NOR exposure could lead to immunotoxicity in crayfish (3 μg/L) [17] and common carp (1 mg/L) [10,18], as well as developmental neurotoxicity and apoptosis in zebrafish embryos (600–1200 mg/L) [19]. Specifically, studies have reported the up-regulation of neurodevelopment-related genes (enolase 2 (*Eno2*) and sex determining region Y-box 2 (*Sox2*)) and elevated apoptotic signals, including activated Caspase 3 (*Cas3*) and an increased the expression ratio of bcl2-associated X protein (*Bax*) to B-cell lymphoma 2 (*Bcl2*), in the brain of exposed embryos [19]. Furthermore, as a representative β-diketone antibiotic (DKA), NOR is also associated with genotoxic effects on fish, such as DNA damage and aberrant DNA hypermethylation [20,21]. While the developmental toxicity, apoptosis, oxidative stress, immunotoxicity, genotoxicity and neurotoxicity of NOR have been relatively well-characterized in aquatic animals [22,23,24], the responses of non-target aquatic animals to its widely used derivative, NOR-N, remain largely unexplored.

The zebrafish is not only a popular ornamental fish but also a favored model organism, possessing a well-characterized genome that enables the investigation of toxicity mechanisms of drugs (e.g., antibiotics) on its physiological functions [25,26]. For instance, FQ antibiotics are known to provoke cardiac functional abnormalities and cardiovascular toxicity in zebrafish, and the intensity of these toxic responses shows a positive relationship with the antibiotic exposure dose [25]. Both chronic exposure to low concentrations and acute exposure to high levels of FQs have been shown to impair the zebrafish motor system, including cartilage tissues and skeletal muscle [27]. Studies have also reported that FQ antibiotics can trigger oxidative stress, epigenetic effects and immunotoxicity in zebrafish [28,29]. Moreover, apoptosis, developmental neurotoxicity and DNA methylation have been recognized as core mechanisms underlying drug-induced toxicity in zebrafish embryos [30,31,32]. For example, apoptosis functions as a cellular response mechanism to exogenous stress. Prior investigations have documented that chemical exposures can trigger apoptotic cell death in zebrafish embryos, accompanied by elevated activities of Cas3 and caspase 9 (Cas9), as well as altered expression levels of apoptosis-related genes such as *Cas9*, tumor protein p53 (*P53*) and p53 up-regulated modulator of apoptosis (*Puma*) [31,33,34,35]. Furthermore, toxicity investigations carried out on zebrafish embryos have revealed that the expression profiles of *Sox* genes (e.g., *Sox2*, *Sox3* and *Sox19a*) can serve as effective indicators for the rapid assessment of potential developmental neurotoxicity caused by environmental toxicant stress [19,36]. Additionally, DNA methylation, a key epigenetic mechanism governing gene expression across all eukaryotic kingdoms, serves a vital function in mediating the defense response to environmental stressors throughout organismal development [37]. Alterations in the mRNA expression of DNA methyltransferase (*Dnmts*) genes, such as *Dnmt1* and *Dnmt3*, in zebrafish are now regarded as valuable biomarkers for signaling early-stage abnormal DNA methylation triggered by environmental stimuli, including drug abuse or toxins [32,35,38]. Research indicates that FQ antibiotic-induced oxidative stress may alter the action of *Dnmts*, thereby triggering hypermethylation in zebrafish larvae [28].

In our previous study, we established five NOR-N exposure concentrations (0.002, 0.2, 1, 5, and 25 mg/L) to evaluate the toxicological impacts of NOR-N on zebrafish embryos over a 96 h post-fertilization (hpf) exposure period. The selected exposure concentrations and durations reflected a combination of relevant exposure scenarios: measured environmental residues of NOR, previously documented effect levels of NOR associated with adverse organismal outcomes, and typical treatment durations used in aquaculture with NOR N. This experimental design demonstrated that NOR-N induced immunotoxicity, oxidative stress and developmental toxicity in zebrafish embryos [39]. Building on these preliminary findings, the present study further investigates NOR-N’s impact on apoptosis, developmental neurotoxicity, and DNA methylation in zebrafish embryos, with the goal of gaining a more thorough comprehension of the response mechanisms of fish to this drug. The specific objectives were: (1) to assess apoptotic responses through multiple indicators, including apoptotic cell death, two key caspase activities (Cas3 and Cas9) and the expression patterns of eight apoptosis-related genes (*P53*, *Bax*, *Puma*, *Apaf1* (apoptotic protease activating factor-1), *Mdm2* (murine double minute 2), *Bcl2*, *Cas3* and *Cas9*); and (2) to evaluate the transcriptional changes in three neurodevelopment-associated genes (*Sox2*, *Sox3* and *Sox19a*) and seven *Dnmts* (e.g., *Dnmt1*, *Dnmt3a1*, *Dnmt3a2*, *Dnmt3b1*, *Dnmt3b2*, *Dnmt3b3* and *Dnmt3b4*) in NOR-N-exposed zebrafish larvae. The findings are expected to provide insights for guiding the safe application of NOR-N in fish farming and assessing its potential ecological risks to aquatic ecosystems.

## 2. Materials and Methods

### 2.1. Zebrafish Maintenance

Healthy adult zebrafish, with a mean weight of 0.46 ± 0.08 g and a standard length of 3.58 ± 0.12 cm, were sourced from the Pearl River Fisheries Research Institute (Guangzhou, China). These zebrafish were maintained in an automated rearing system filled with dechlorinated tap water under controlled environmental conditions: temperature 28 ± 1 °C, pH 7.2 ± 0.1, dissolved oxygen 6.3 ± 0.2 mg/L, total water hardness 122.7 ± 3.5 mg/L as CaCO_3_, and a 14 h light/10 h dark photoperiod. They were provided with brine shrimp as feed twice per day. Prior to spawning induction, adult zebrafish (1 female per 2 males) were housed in separate compartments of spawning tanks overnight. The next morning, spawning was triggered by exposure to full-spectrum LED light (1000 Lux) for 3–4 h; embryos were then gathered within 30 min post-spawning and washed with the system water. At 4 hpf, fertilized embryos exhibiting normal development were identified under an Olympus microscope (Tokyo, Japan) and used in the exposure assay. The animal experimental protocol used in this research was examined and granted approval by Jiangxi Agricultural University’s Committee for the Care and Use of Experimental Animals.

### 2.2. Experimental Design

NOR-N (purity > 98%, obtained from J&K Scientific, Beijing, China) was dissolved in dechlorinated tap water to prepare a stock solution at 1000 mg/L. Test solutions of varying concentrations (0.002, 0.2, 1, 5, and 25 mg/L) were subsequently prepared by performing serial dilutions of this stock solution. The conditions and protocols for exposure were consistent with those described in our prior research [39]. Briefly, the selection of exposure concentrations and durations was based on three key considerations: (1) the concentrations of 0.002, 0.2, 1, and 5 mg/L were chosen to mimic the actual NOR concentrations reported in surface waters and aquaculture ponds, which span 0.03 µg/L to 6.06 mg/L—where 0.002 and 0.2 mg/L correspond to normal environmentally relevant levels, while 1 and 5 mg/L represent exceptional environmental concentrations; (2) the high concentration of 25 mg/L was designated as a biologically effective level, as it has been shown to induce significant adverse effects on organisms in preceding investigations; and (3) the 96 hpf exposure duration was designed to align with the relevant fishery industry guidelines for NOR-N, which specify a recommended oral dosage of 15–30 mg/kg body weight (expressed as NOR equivalents) administered over a 3–5 day period. Sixty normally developing 4 hpf (sphere-stage) embryos were randomly allocated to each treatment. Using a static-renewal exposure protocol, the embryos were maintained in 100 mL glass beakers containing 60 mL of the respective exposure solutions until 96 hpf. Each experimental group, including the dechlorinated tap water control and all NOR-N treatments, was conducted with three replicates. All exposures were conducted under the following conditions: temperature 28 ± 1 °C, pH 7.3 ± 0.1, dissolved oxygen 6.3 ± 0.2 mg/L, total water hardness 129.4 ± 4.1 mg/L as CaCO_3_, and a 14 h light/10 h dark cycle. Daily maintenance involved replacing half of the test solution in each beaker to maintain stable exposure concentrations, and monitoring embryo survival twice daily, with immediate removal of any dead individuals. Embryo mortality across experimental groups was low (5–8%), providing a viable sample size of larvae in each group for the following analytical stages. Upon termination of the experiment at 96 hpf, samples were collected from three independent replicate beakers per group (*n* = 3). Approximately five newly hatched larvae per beaker were sampled for the analysis of apoptotic cells. Additionally, fifteen larvae per beaker were pooled as a single sample for caspase activity assays, and another fifteen were pooled for gene transcription analysis.

### 2.3. Measurements of Apoptotic Cells

Apoptotic cells in live zebrafish larvae were visualized using acridine orange (AO), a fluorescent dye that selectively stains nucleic acids and serves as a marker for apoptosis [40]. Following three rinses with PBS, the larvae were placed in 5 μg/mL AO at 28 °C in the dark for 30 min. After staining, they were subjected to another three PBS washes [41], briefly anesthetized with 0.03% MS-222, and immediately examined under an Olympus fluorescent microscope (Japan). The semi-quantitative analysis of apoptotic cells was conducted by two blinded researchers independently, yielding consistent results between observers.

### 2.4. Determination of Caspase Enzymatic Activity

Upon termination of the experiment at 96 hpf, a pool of fifteen larvae per replicate (*n* = 3) was homogenized on ice in 200 µL of the provided kit lysis buffer. The homogenate underwent centrifugation at 10,000× *g* for 15 min at 4 °C, and the resulting supernatant was collected to assay Cas3 and Cas9 activities, as well as the total protein content, using commercial kits (Beyotime, Shanghai, China) according to the manufacturer’s protocols. Moreover, to enhance clarity and facilitate direct comparison, caspase activities (expressed in % of control) were calculated as a percentage in relation to the control group.

### 2.5. Gene Expression Analysis

Trizol reagent (Invitrogen, Carlsbad, CA, USA) was used to isolate total RNA from pools of 15 zebrafish larvae. The integrity and concentration of RNA were, respectively, validated by 1% agarose gel electrophoresis and UV spectrophotometry. Subsequently, 500 ng of total RNA was transcribed reversely into cDNA using the RT reagent Kit (PrimeScript^®^, Takara Biotechnology, Dalian, China), and the produced cDNA was preserved at −20 °C for subsequent real-time quantitative PCR (qPCR) analysis.

Expression analysis of the *P53*, *Bax*, *Puma*, *Apaf1*, *Mdm2*, *Bcl2*, *Cas3*, *Cas9*, *Sox2*, *Sox3*, *Sox19a*, *Dnmt1*, *Dnmt3a1*, *Dnmt3a2*, *Dnmt3b1*, *Dnmt3b2*, *Dnmt3b3* and *Dnmt3b4* genes was performed via qPCR. Reactions were performed in a 20 μL mixture including SYBR Green Premix (Takara Biotechnology, China) and gene-specific primers (Table 1), employing a qPCR System (ABI 7500, Applied Biosystems, Foster City, CA, USA). The cycling program was: 95 °C for 30 s, followed by 40 cycles of 95 °C for 10 s and 60 °C for 34 s, with a melting curve step to verify amplicon specificity. The primers demonstrated amplification efficiencies ranging from 90% to 105%, with melting curve analyses showing a single sharp peak specific to the target amplicon. All reactions were run in triplicate. Relative gene expression was normalized to β-actin and quantified using the 2^−△△Ct^ method.

### 2.6. Statistical Analysis

Data from all assays were expressed as mean ± standard deviation (SD). To compare treatment groups with the control, one-way analysis of variance (ANOVA) was performed, followed by Dunnett’s post hoc test for multiple comparisons (SPSS 22.0, IBM, Chicago, IL, USA). Levene’s test was conducted to verify variance homogeneity prior to parametric analysis. Significance was accepted at *p* < 0.05.

## 3. Results

### 3.1. Observation of Apoptotic Cells in Live Zebrafish Larvae Following NOR-N Exposure

**Figure 1:**
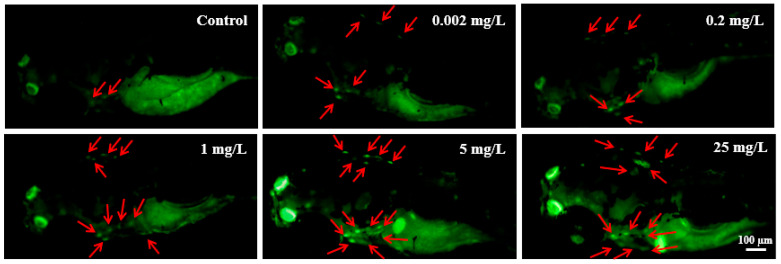
Apoptotic cells in live zebrafish larvae following exposure to different doses of NOR-N. Red arrows indicate apoptotic cells accumulated in the brain and heart regions of NOR-N exposed larvae.

AO staining of zebrafish larvae at 96 hpf revealed a dose-dependent increase in the number of apoptotic cells within the brain and heart regions. This increase was particularly notable in larvae exposed to 5 and 25 mg/L NOR-N (Figure 1).

### 3.2. Changes in Caspase Enzymatic Activity in Zebrafish Larvae Exposed to NOR-N

**Figure 2:**
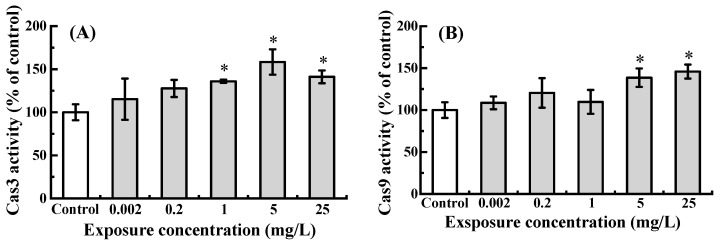
Cas3 (**A**) and Cas9 (**B**) activities in zebrafish larvae following embryonic exposure to NOR-N. Activity levels are expressed as a percentage of the control. Values are presented as mean ± SD (*n* = 3; 15 larvae for each *n*). Asterisks (*) indicate significant differences from the control (*p* < 0.05).

Exposure to NOR-N led to a dose-dependent increase in the activities of Cas3 and Cas9 (Figure 2). While the lowest dose (0.002 mg/L) only caused a non-significant rise (*p* > 0.05) compared to the control, a statistically marked induction was observed at higher doses (≥1 mg/L for Cas3 and ≥5 mg/L for Cas9, *p* < 0.05).

### 3.3. Responses of Apoptosis-Related Gene Expression in Zebrafish Larvae to NOR-N Exposure

**Figure 3:**
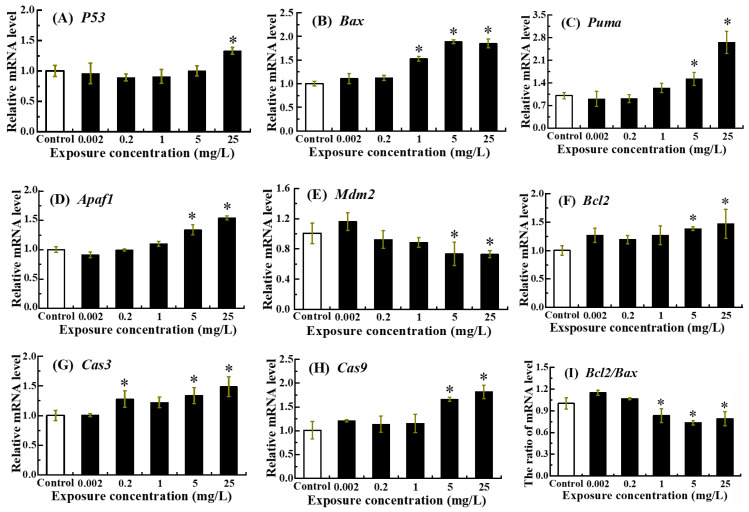
Expression levels of apoptosis-related genes in zebrafish larvae following embryonic exposure to NOR-N. Relative mRNA levels of (**A**) *P53*, (**B**) *Bax*, (**C**) *Puma*, (**D**) *Apaf1*, (**E**) *Mdm2*, (**F**) *Bcl2*, (**G**) *Cas3*, (**H**) *Cas9* and (**I**) *Bcl2/Bax* ratio were assessed. Values are presented as mean ± SD (*n* = 3; 15 larvae for each *n*). Asterisks (*) indicate significant differences from the control (*p* < 0.05).

The expression levels of *P53*, *Bax*, *Puma*, *Apaf1*, *Mdm2*, *Bcl2*, *Cas3*, *Cas9* and *Bcl2/Bax* ratio were profiled in zebrafish embryos following exposure to NOR-N until 96 hpf (Figure 3).

Relative to the control group, a significant increase in *P53* mRNA was detected exclusively in the 25 mg/L treatment group (*p* < 0.05) (Figure 3A). Transcriptional levels of *Bax*, *Puma*, *Apaf1*, *Bcl2*, *Cas3* and *Cas9* exhibited a general dose-dependent upward trend, with marked elevations observed following treatment with 5 and 25 mg/L NOR-N (*p* < 0.05) (Figure 3B–D,F–H). In contrast, both *Mdm2* mRNA expression and the *Bcl2/Bax* ratio displayed clear dose-dependent reductions (Figure 3E,I). Specifically, *Mdm2* transcripts were significantly decreased in the 5 and 25 mg/L treatment groups (*p* < 0.05), while the *Bcl2/Bax* ratio showed significant declines in the 1, 5 and 25 mg/L treatment groups (*p* < 0.05).

### 3.4. Neurodevelopment-Related Gene Expression Patterns in Zebrafish Larvae Under NOR-N Exposure

**Figure 4:**
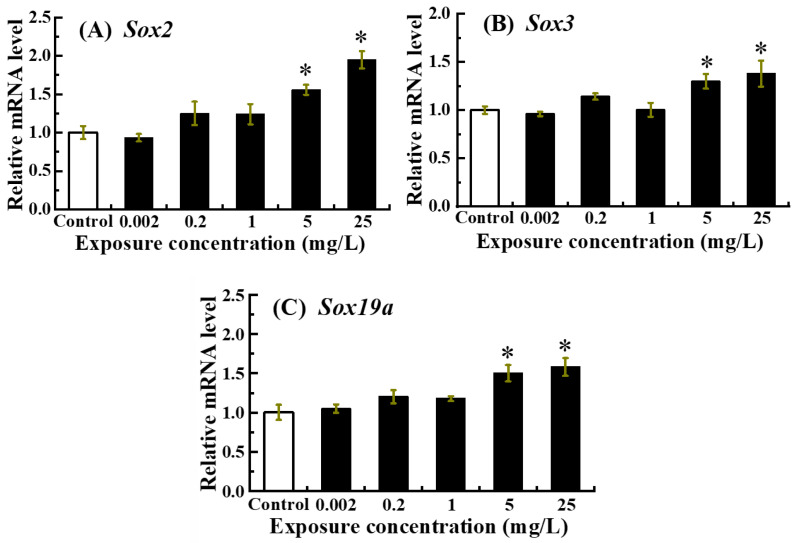
Relative expression levels of neurodevelopment-related genes in zebrafish larvae following embryonic exposure to NOR-N. Transcript levels of (**A**) *sox2*, (**B**) *sox3*, and (**C**) *sox19a* were measured. Values are presented as mean ± SD (*n* = 3; 15 larvae for each *n*). Asterisks (*) indicate significant differences from the control (*p* < 0.05).

The mRNA expression of neurodevelopment-related genes including *Sox2*, *Sox3* and *Sox19a* was presented in Figure 4. *Sox2*, *Sox3* and *Sox19a* mRNA expression levels exhibited a gradual upward trend as the exposure dose of NOR-N increased, with significant up-regulation detected in the 5 and 25 mg/L NOR-N treatment groups (*p* < 0.05).

### 3.5. Transcriptional Responses of DNA Methylation-Associated Genes in Zebrafish Larvae to NOR-N Exposure

**Figure 5:**
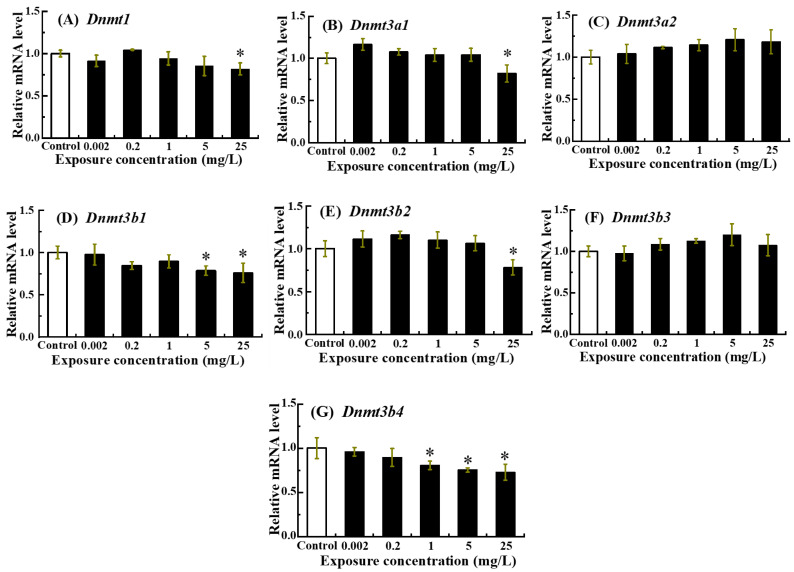
Transcription levels of DNA methylation-associated genes in zebrafish larvae following embryonic exposure to NOR-N. Relative mRNA levels of (**A**) *Dnmt1*, (**B**) *Dnmt3a1*, (**C**) *Dnmt3a2*, (**D**) *Dnmt3b1*, (**E**) *Dnmt3b2*, (**F**) *Dnmt3b3* and (**G**) *Dnmt3b4* were measured. Values are presented as mean ± SD (*n* = 3; 15 larvae for each *n*). Asterisks (*) indicate significant differences from the control (*p* < 0.05).

The transcriptional responses of DNA methylation-associated genes to NOR-N exposure were shown in Figure 5. A dose-dependent decrease was observed for *Dnmt1*, *Dnmt3a1*, *Dnmt3b1*, *Dnmt3b2* and *Dnmt3b4*, with expression reaching its lowest point at 25 mg/L (*p* < 0.05) (Figure 5A,B,D,E,G). Conversely, *Dnmt3a2* and *Dnmt3b3* mRNA levels showed no significant variation compared to the control (*p* > 0.05) (Figure 5C,F).

## 4. Discussion

Prior investigations have indicated that NOR possesses the ability to trigger developmental toxicity, apoptosis, oxidative stress, immunotoxicity, genotoxicity and neurotoxicity in non-target aquatic organisms, such as various fish species [13,16,19,22]. Consequently, considering that NOR-N serves as an adduct and substitute for NOR, the potential harmful impacts of this substance on aquatic organisms warrant attention. Our previous study found that NOR-N exposure could induce immunotoxicity, oxidative stress and developmental toxicity in zebrafish embryos [39]. Nevertheless, to gain a more profound understanding of the fish response mechanisms under NOR-N exposure, it is worthwhile to conduct further investigations into the additional endpoints, including apoptosis, developmental neurotoxicity, and DNA methylation in zebrafish embryos.

Apoptosis serves as an essential mediator of growth and development in organisms including fish, and it usually participates in response to diverse environmental stressors during the early development stage [30,40,42,43]. In the current investigation, exposure to NOR-N led to a dose-dependent increase in apoptotic cells within the brain and heart regions of live zebrafish larvae, indicating that significant apoptosis induction occurred, particularly in the high dose groups (≥5 mg/L).

As is well known, the mitochondria-dependent apoptotic pathway serves as a key regulatory mechanism for cell fate [44]. *P53* not only promotes the mitochondrial release of cytochrome c into the cytosol through the activation of pro-apoptotic genes, such as *Puma* and *Bax*, but also directly upregulates the transcription of its key negative regulator, *Mdm2* [45,46]. Once in the cytoplasm, cytochrome c forms a complex with *Apaf1*, and this complex induces the sequential activation of *Cas9* and then *Cas3* [47]. Additionally, during apoptosis, a decrease in the *Bcl-2/Bax* ratio is a critical event that facilitates cytochrome c release from mitochondria, serving as a decisive indicator of cell death commitment [48]. The current study revealed that exposure to NOR-N caused a dose-dependent increase in *P53*, *Bax*, *Puma*, *Apaf1*, *Cas3* and *Cas9* mRNA levels, along with elevated enzymatic activities of Cas3 and Cas9. The parallel elevation in both transcript abundance and catalytic function of these caspases implies that their activity is regulated at the transcriptional level via an autogenous mechanism, thereby implicating the caspase signaling pathway in NOR-N-induced apoptosis in zebrafish larvae [48]. Concurrently, a decrease was observed in anti-apoptotic indicators, including *Mdm2* expression and the *Bcl2/Bax* ratio in the high dose groups (≥5 mg/L). As evidenced by severe apoptosis in *Mdm2*-knockdown embryos, *P53* has been shown to be essential for DNA damage-induced apoptosis in developing zebrafish following environmental insults (e.g., irradiation) [49]. Consistent with this, we found that NOR-N exposure triggered a marked increase in *P53* expression and a concurrent significant decrease in *Mdm2* expression, indicating that both genes play a role in mediating the apoptotic response. Overall, these results implied that high doses of NOR-N (5 and/or 25 mg/L) activated the mitochondria-dependent pathway to induce apoptotic cell death in zebrafish larvae. Consistent with this, prior investigations have indicated that antibiotics, fungicides and other xenobiotics induced apoptosis in zebrafish larvae, which was associated with the activation of Cas3, the induction of mRNA levels of *P53*, *Bax*, *Apaf1*, *Cas3* and *Cas9*, and the reduction in the *Bcl2/Bax* ratio [19,31,50].

The proper expression of *Sox* genes is pivotal for normal nervous system formation in zebrafish embryos, and any alteration may result in adverse developmental outcomes [36]. Among *Sox* genes, *Sox2* is critical for maintaining pluripotency, cellular differentiation, and neuronal formation in zebrafish [51], while *Sox3*, often alongside *Sox1* and *Sox2*, acts as a key transcription factor in early vertebrate neurodevelopment and neural stem cell maintenance [52]. *Sox19a*, recognized as one of the earliest markers of the central nervous system of vertebrates, further underscores the importance of this gene family [53]. Consistent with these roles, knockdown of *Sox19a*, *Sox2* or *Sox3* in zebrafish embryos led to severe central nervous system abnormalities [54]. Prior investigations have also indicated that exogenous chemicals could disrupt nervous system development by interfering with the transcription of *Sox* genes [32,55,56]. Importantly, *Sox2* is re-expressed in mature neural tissues post-injury and is essential for initiating repair and neural plasticity mechanisms, often as part of a glial or progenitor cell response aimed at counteracting damage [57]. This implied that *Sox* expression may represent a double-edged sword, serving both as a marker of neurotoxic insult and a potential indicator of the brain’s innate repair mechanisms being activated. In the current study, NOR-N exposure exhibited a dose-dependent increase in *Sox2*, *Sox3*, and *Sox19a* mRNA levels in zebrafish larvae. This up regulation may reflect an attempt by the larvae to mobilize intrinsic resilience mechanisms against the neurotoxic challenge posed by high doses of NOR-N (5 and 25 mg/L). These findings align with earlier reports showing that NOR caused developmental neurotoxicity in larval zebrafish, as evidenced by up-regulating *Sox2* and *Eno2* (a mature neuron marker) gene expressions [19].

Additionally, it is noteworthy that the developmental neurotoxicity caused by NOR-N in this study was evidenced by the obvious apoptotic cell death in the brain region of larval zebrafish, which is recognized as a late-stage neurotoxicity biomarker [58]. The parallel increases in brain apoptosis and the transcript levels of *Sox2*, *Sox3*, and *Sox19a* suggested that the NOR-N-induced overexpression of these *Sox* genes might represent a key molecular event underlying NOR-N-induced developmental neurotoxicity. Given these findings, the potential hazards of NOR-N to the developing nervous system in fish warrant serious consideration, and further investigation is needed to fully elucidate the precise response mechanisms in fish induced by this drug.

DNA methylation functions as a key epigenetic mechanism implicated in a variety of biological processes such as genomic imprinting, X-chromosome inactivation and cellular differentiation. Alterations in DNA methylation represent a major non-genotoxic effect caused by exogenous toxic chemicals [37]. This process is regulated by DNA methyltransferases, which establish and maintain genome-wide methylation patterns [59]. As is widely recognized, *Dnmt1* functions to maintain the global DNA methylation patterns during DNA replication, whereas *Dnmt3b* and *Dnmt3a* regulate de novo methylation, critical for establishing new patterns during early embryogenesis [60,61]. In the current research, following NOR-N exposure, the expression of *Dnmt3a2* and *Dnmt3b3* remained unchanged, whereas the mRNA expression levels of *Dnmt1*, *Dnmt3a1*, *Dnmt3b1*, *Dnmt3b2*, and *Dnmt3b4* were downregulated at 5 and/or 25 mg/L. This bifurcated pattern suggests a targeted cellular adaptation, in which the sustained expression of *Dnmt3a2* and *Dnmt3b3* may serve a compensatory or housekeeping role, ensuring precise methylation at genomic loci critical for cellular homeostasis or for the controlled execution of apoptosis [62]. Conversely, the broad suppression of multiple *Dnmts* could facilitate a programmed reduction in global DNA methylation, a change often linked to the activation of stress response and pro apoptotic genes [63]. Together, these results imply suggested that high doses of NOR-N might disrupt epigenetic regulation by impairing both the DNA methylation maintenance and de novo methylation processes during early zebrafish development. This finding aligns with previous reports that attribute pollutant-induced alterations in DNA methylation to the dysregulated expression of *Dnmt3* and *Dnmt1* genes [32,38,64]. For example, Pb exposure has been shown to decrease the expression of *Dnmt3b* and *Dnmt1* and the level of global DNA methylation in zebrafish [35,65]. Similarly, Benzo[a]pyrene was observed to generally suppress *Dnmt* expression and lower global methylation during the development of zebrafish [66], and atrazine exposure was demonstrated to inhibit both the activity and expression of *Dnmts*, leading to reduced global methylation [67]. These examples illustrate that high doses of NOR-N might similarly regulate global DNA methylation patterns by down-regulating *Dnmt* expression in larval zebrafish. However, the extent to which NOR-N exposure affects genome-wide methylation or specific gene-level methylation in fish remains unclear, necessitating further research to clarify the detailed mechanisms for epigenetic disruption in fish under NOR-N exposure.

This study demonstrated that exposure to high doses (5 and/or 25 mg/L) of NOR-N led to apoptosis, developmental neurotoxicity, and aberrant DNA methylation in zebrafish larvae at 96 hpf. We recognize certain limitations in translating these findings to environmental contexts. First, current environmental monitoring focuses on the metabolite NOR, with no available data on environmental levels of the parent compound NOR-N. Consequently, our exposure concentrations were based on NOR data and may not accurately reflect real-world NOR-N levels, and we could not distinguish between the biological effects of NOR-N and its active metabolite, NOR. Second, while the zebrafish embryo-larval model is a robust and standardized system for early-life toxicity screening, our conclusions are inherently limited to this specific model and developmental stage. To address these gaps, future work should focus on several key directions. Environmental monitoring must be expanded to specifically quantify NOR-N in aquatic environments, establishing realistic exposure ranges for toxicological testing. Comparative toxicology studies are needed to disentangle the distinct effects of NOR-N and NOR through parallel exposure experiments, supported by analytical methods to track their fate and bioaccumulation. Expanding biological models is crucial; incorporating additional test species (e.g., Daphnia magna or commercially relevant fish) and life stages (e.g., adults) would better define ecological risks and sensitive windows of toxicity. Finally, mechanistic investigations using in vitro systems—such as piscine (e.g., ZFL) or mammalian (e.g., HEK293T) cell lines—could efficiently elucidate direct molecular actions (e.g., on Dnmt activity) [68,69] and provide preliminary insight into potential human health implications, especially given exposure routes via water and food chains.

## 5. Conclusions

In conclusion, this study showed that exposing zebrafish larvae to high doses of NOR-N (5 and/or 25 mg/L) triggered apoptotic cell death, enhanced Cas9 and Cas3 activities, altered transcript levels of mitochondria-dependent apoptotic pathway genes, up-regulated key neurodevelopmental genes, and suppressed genes associated with DNA methylation. These results indicated that NOR-N induced apoptosis, developmental neurotoxicity, and aberrant DNA methylation in zebrafish larvae. The findings offer valuable insights into the response mechanisms of fish to NOR-N exposure and provide a scientific basis for guiding NOR-N’s safe application in fish farming. Nevertheless, deepening our understanding of NOR-N’s molecular and cellular toxic mechanisms requires additional studies employing diverse aquatic species and in vitro models.

## Figures and Tables

**Table 1:** Sequences of primers used for real-time quantitative PCR.

Target Gene	Forward Primer (from 5′ to 3′)	Reverse Primer (from 5′ to 3′)	Amplicon		References
			Size (bp)	Tm (°C)	
*P53*	GGGCAATCAGCGAGCAAA	ACTGACCTTCCTGAGTCTCCA	197	84	[40]
*Puma*	TGGAAAGCAGAGTGGACGAA	GATGGCAGGGCTGGATGA	108	86	[40]
*Mdm2*	AAGCAGTGATCCTGAGAGTCC	ATCCGAAGACTCGCTGTTC	157	85	[40]
*Bax*	GGCTATTTCAACCAGGGTTCC	TGCGAATCACCAATGCTGT	197	85	[40]
*Bcl2*	TCACTCGTTCAGACCCTCAT	ACGCTTTCCACGCACAT	235	85	[40]
*Apaf1*	TTCTACAGTAAACGCCCACC	TATCTAGTATTTCCCCATATTCC	205	83	[40]
*Cas3*	CCGCTGCCCATCACTA	ATCCTTTCACGACCATCT	129	83	[40]
*Cas9*	AAATACATAGCAAGGCAACC	CACAGGGAATCAAGAAAGG	103	83	[40]
*Sox2*	CTCGGGAAACAACCAGAAAA	TCGCTCTCGGACAGAAGTTT	171	86	[36]
*Sox3*	ACCGAGATTAAAAGCCCCAT	TTGCTGATCTCCGAGTTGTG	182	88	[36]
*Sox19a*	TGTCAACAGCAACAACAGCA	GTTGTGCATTTTGGGGTTCT	126	84	[36]
*Dnmt1*	GGGCTACCAGTGCACCTTTG	GATGATAGCTCTGCGTCGAGTC	76	85	[38]
*Dnmt3a1*	GCTAAGTTTGGTAAAGTGCGG	GGATGTCCTCCTTATCATTCA	103	81	[38]
*Dnmt3a2*	TAGGAAAGGCTTGTTTGAGGG	GCGTGAGATGTCTTTCTTGTC	157	86	[38]
*Dnmt3b1*	CGTGTTGCCAAGTTCGGG	ATCCTCTTTGCCATTCATCA	105	82	[38]
*Dnmt3b2*	CGCTACATTGCCTCTGAGA	GCCAGATGTTTCCTAGTGATG	104	82	[38]
*Dnmt3b3*	GCTCAGGTGCTGCTTTTTGTC	TTTTTGAATCTGTGCTTTGCTG	152	79	[38]
*Dnmt3b4*	CAACACACTGACAACATCAAGAG	TCAATGACATCTAGCATCTCTGG	134	81	[38]
*β-actin*	CGAGCAGGAGATGGGAACC	CAACGGAAACGCTCATTGC	102	85	[40]

## Data Availability

The original contributions presented in this study are included in the article. Further inquiries can be directed to the corresponding authors.

## Abbreviations

The following abbreviations are used in this manuscript:

NOR	Norfloxacin
FQ	Fluoroquinolone
NOR-N	Norfloxacin nicotinate
Gpx	Glutathione peroxidase
CAT	catalase
SOD	superoxide dismutase
*Eno2*	enolase 2
*Sox*	sex determining region Y-box
*Sox2*	sex determining region Y-box 2
Cas3	Caspase 3
*Bax*	bcl2-associated X protein
*Bcl2*	B-cell lymphoma 2
DKA	β-diketone antibiotic
Cas9	caspase 9
*P53*	tumor protein p53
*Puma*	p53 up-regulated modulator of apoptosis
*Dnmts*	DNA methyltransferase
*Apaf1*	apoptotic protease activating factor-1
*Mdm2*	murine double minute 2
hpf	hours post-fertilization
AO	acridine orange
qPCR	real-time quantitative PCR
SD	standard deviation
ANOVA	one-way analysis of variance
